# Generalized overview infographic: a customizable library instructional material on the NIH Data Management and Sharing Policy

**DOI:** 10.5195/jmla.2024.1867

**Published:** 2024-04-01

**Authors:** Katy [Katherine] Smith

**Affiliations:** 1 katy.smith@slu.edu, Health Sciences Librarian, Medical Center Library, Saint Louis University, St. Louis, MO

**Keywords:** NIH Data Management and Sharing Policy, Infographic, Flow-chart, Decision Tree, Canva

## Abstract

The Generalized Overview of the NIH Data Management and Sharing Policy Effective 2023.01.15 (Generalized Overview) is an instructional material that provides a basic, clear, and linear understanding of the NIH policy and its requirements. While not developing or utilizing new technology, the Generalized Overview is innovative and notable for creatively using a freely available graphic design tool to translate government policy language into an accessible and understandable infographic that can disseminate important information about the NIH DMS Policy needed by researchers and by those who support them. Shared via a Creative Commons license, others may fully adapt the infographic or may simply add their own institutional contact information. The Generalized Overview can be used by any who find themselves responsible for publicizing and/or teaching the NIH Data Management and Sharing Policy at their respective libraries and institutions. It is intended for educational purposes only and should not be used as a substitute for official guidance from the NIH.

## CONTEXT, AIMS, AND SIGNIFICANCE OF THE VIRTUAL PROJECT

As library services evolve to meet the expanding user needs related to publicly mandated and/or privately required data management and sharing policies, librarians, particularly many-hatted liaison librarians, may find themselves with new responsibilities supporting data services. While some librarians may have previous knowledge and experience in research data management, others may have only a limited working knowledge. Attempting to publicize and teach policies while simultaneously learning said policies—as well as data services in general—can be overwhelming. However, other knowledge and/or past experiences can prove beneficial by enabling the application of different approaches to library instruction and services, as in the following development of a simple instructional visual representation of the 2023 NIH Data Management and Sharing Policy (NIH DMS Policy) as released via the Final NIH Policy for Data Management and Sharing [1] and the Supplemental Information to the NIH Policy for Data Management and Sharing: Elements of an NIH Data Management and Sharing Plan [2].

Joining a medical center library in November 2021, the author found herself responsible for data services (among other traditional liaison areas) with little data sciences background beyond that used with personal dissertation research data. After finding encouragement and coaching from newly found brilliant and gracious colleagues in the Medical Library Association Data Caucus, the author utilized past public and secondary school/junior college library experiences in information literacy instruction to effectively and efficiently visualize and to share the basic information of the NIH DMS Policy within an infographic. While not developing or utilizing new technology, the Generalized Overview is innovative and notable for creatively using a freely available graphic design tool to translate government policy language into an accessible and understandable infographic that can quickly—and more importantly, accurately—disseminate the important information needed by researchers and by those who support them.

## BRIEF DESCRIPTION OF THE VIRTUAL PROJECT

**Figure 1:**
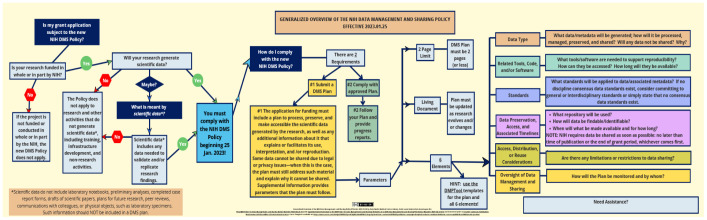


Inspired by a fellow data librarian's example of color-coding pertinent portions of the DMS Policy [3], the author initially sought to solidify a personal basic understanding of the policy by breaking down the policy language and mind-mapping it within Canva, a freely available online graphic design tool. After a few false starts, Canva was further used to transform the policy into an easy-to-follow flow chart infographic—the Generalized Overview—providing a basic, linear, and clear understanding of the NIH DMS Policy and of its requirements. As illustrated in Figure 1, the infographic begins with addressing the question of whether or not one's grant application is subject to the NIH DMS Policy, then leads the viewer via a flow-chart/decision tree process through the two compliance requirements (submitting a plan and complying with said plan) and the parameters of the plan (two-page limit, evolving documentation, and the six required elements: data type; related tools, code, and/or software; standards; data preservation, access, and timelines; access, distribution, or reuse considerations; and oversight).

## TECHNOLOGY USED

Canva [4] is an online tool that simplifies graphic design with an intuitive platform that can be used via any web browser as well as via an Android/iOS supported mobile application. Free to use after registering with an email account, Canva provides a straightforward drag-and-drop editable interface to create or to customize 100+ design types (such as posters, slide decks, and infographics) using 250,0000+ templates and over a million free photos and/or graphic elements. Users can also freely create designs with uploaded personal content. With 5GB of cloud storage, users can share designs, invite others to view and to edit, and can order printable products featuring their designs. Canva Pro, at $119.99 per year, provides additional features, including expanded collections of templates and graphics, stock audio and video, AI-based tools, and 24/7 customer support; however, the elements used to create the Generalized Overview were all available within the free version.

## ADVANTAGES, LIMITATIONS, AND IMPACT

After basing a brief on-campus overview instruction session around the Generalized Overview and describing the successful instructional use of the infographic to colleagues, the author received multiple requests to share it. It has since been well received by librarians, researchers, and other stakeholders at and beyond the author's library and institution. Licensed under Creative Commons, others may fully adapt the infographic or may simply add their own institutional contact information before sharing with their communities via Canva, Microsoft PowerPoint, or .pdf versions—each downloadable via a Library Guide [5]. Although limited in scale and providing only the bare basics of the NIH DMS Policy, the Generalized Overview can be efficiently and effectively used by any who find themselves responsible for learning, publicizing, and/or teaching the NIH DMS Policy at their respective libraries and institutions. The Generalized Overview is intended for educational purposes only and does not act as a substitute for official guidance from the NIH.

## AUTHOR CONTRIBUTIONS

The author confirms sole responsibility for the following: project conception and design, writing, reviewing, and editing.

## References

[1] United States National Institutes of Health. Final NIH policy for data management and sharing: NOT-OD-21-013 [Internet]. [29 Oct 2020; cited 20 Oct 2023]. Available from: https://grants.nih.gov/grants/guide/notice-files/NOTOD-21-013.html.

[2] United States National Institutes of Health. Supplemental information to the NIH policy for data management and sharing: Elements of an NIH data management and sharing plan: NOT-OD-21-014 [Internet]. [29 Oct 2020; cited 20 Oct 2023]. Available from: https://grants.nih.gov/grants/guide/notice-files/NOTOD-21-014.html.

[3] Exner, N. Preparing your workshops on the new NIH data management and sharing plans-DMSPs [Internet]. MLA Webinars. [28 June 2022; cited 20 Oct 2023].

[4] Canva. [Internet]. [2024; cited 20 Oct 2023]. Available from: http://www.canva.com.

[5] Smith, K. Customizable Version of Generalized Overview Infographic. NIH Data Management and Sharing Policy. [Internet]. Research Guides. Saint Louis University Medical Center Library. [20 Oct 2023; cited 20 Oct 2023]. Available from: https://libguides.slu.edu/NIH_DMSP/Generalized_Overview_Infographic.

